# Knowledge level of diagnostic procedures and risk factors for oral cancer among oral healthcare providers in Germany

**DOI:** 10.1186/s12903-025-06048-5

**Published:** 2025-05-02

**Authors:** Rieke Scharbrodt, Sarah Habig, Michael Kalab, Eva Baumann, Lisa Felgendreff, Astrid Dempfle, Katrin Hertrampf

**Affiliations:** 1 Department of Oral and Maxillofacial Surgery, University Hospital Schleswig-Holstein, Kiel Campus, Arnold-Heller Street 3, Building B, Kiel, 24105 Germany; 2 Institute of Medical Informatics and Statistics, Kiel University, University Hospital Schleswig-Holstein, Kiel Campus, Brunswiker Street 10, Kiel, 24105 Germany; 3 Department of Journalism and Communication Research, Hannover University of Music, Drama, and Media, 12 Expo Plaza, Hannover, 30539 Germany

**Keywords:** Oral cancer, Prevention, Knowledge, Opinion, Continuing education, Online survey, Dentist, Maxillofacial surgeon, Germany

## Abstract

**Background:**

Oral cancer is an underestimated and growing public health problem. The majority of cases are diagnosed at a late stage, even though oral cancer can be detected early by routine visual and tactile examination. Dental healthcare providers thus play a vital role in its early detection. This study assessed dental healthcare providers’ knowledge of diagnostic procedures and risk factors for oral cancer.

**Methods:**

A cross-sectional, observational study was conducted using a validated survey of 38 items focusing on knowledge of diagnostic procedures and risk factors, and questions on continuing education. From October to December 2023, the online survey invitation was sent via all German state Dental Associations to their members. Participants' socio-demographic data and responses in the “knowledge, opinion and continuing education” section were analysed descriptively. Linear regressions assessed the associations between participants’ characteristics or opinions and their knowledge of oral cancer (knowledge score).

**Results:**

Overall, 8,132 participants began the survey. After filtering for reliability and completeness, data from 3,458, 3,472, and 2,933 participants were available for "socio-demographics", "knowledge", and "opinions and continuing education", respectively. Most participants were women (60%), between 30 to 49 years old (48%). The majority knew the most common type of oral cancer and the most common lesions associated with it. Furthermore, almost 89% of the participants were aware that early detection improves survival rates. The main risk factors (tobacco, alcohol, prior oral cancer) were well known. However, the potential for improvement in knowledge of localisation, signs, stage of diagnosis and the specific age group at risk was noted. Fewer years in the dental profession and since attending the last continuing education course were associated with higher levels of knowledge.

**Conclusions:**

Knowledge of localisation, signs, stage of diagnosis, and specific risk groups can be improved. These topics should thus be integral to the dental curriculum and addressed through structured continuing education programmes, as the study’s results suggest that knowledge declines over time without continuing education.

**Supplementary Information:**

The online version contains supplementary material available at 10.1186/s12903-025-06048-5.

## Introduction

Tumours in the oral cavity continue to be underestimated diseases. The estimated incident cases worldwide are over 389,000 per year, with more men affected than women (about 268,999 men vs. 120,847 women [1]. In Europe, 62,103 new cases were reported with a similar gender distribution (41,607 men vs. 20,496 women) [1]. The Eurocare-6 study reported a 5-year survival rate for head and neck cancer of 44% for men and 58% for women for 2020. A further stratification by oral cancer was not performed [2].

In Germany, the estimated number of new oral cavity and pharynx tumour cases was over 13,000 (9,140 men vs 4,050 women) in 2020, with more than 50% located in the oral cavity. Although treatment standards in diagnostics and therapy have continuously improved in recent decades, this has not had a sufficiently positive effect on reducing the mortality rate. The average five-year survival rate was only 52% for men and 64% for women [3].

More than half of those affected only present to a dentist, oral and maxillofacial surgeon or another specialised medical discipline at an advanced stage, as the disease is painless and asymptomatic for a relatively long time [3, 4]. In addition, there is a high potential for displacement of the disease and the possible symptoms, as is also the case with other cancers. This situation necessitates complex treatment after diagnosis, with a negative impact on prognosis. Identifying the tumour earlier would improve the probability of survival [5, 6].

In Germany, those affected often turn first to their dentist or medical specialist, who encounter this type of tumour in their everyday work. However, as this disease is not part of routine dental practice, there might not be sufficient knowledge and training in recognising and making an initial diagnosis [7].

The required standardised visual clinical examination of the oral mucosa offers a form of early detection that is painless, quick, and has no side effects.

International regional studies and our regional German survey results showed that there is potential to improve the knowledge levels of oral cancer and the measures for early detection, and that dentists were uncertain in assessing their levels of knowledge [8–13].

Having this knowledge is important for dentists to carry out the necessary early detection examinations competently and provide patients with adequate information. Studies have shown that targeted training programmes lasting several months can improve knowledge [14–16]. In Germany, based on the results of a formative multi-level evaluation in the federal state of Schleswig–Holstein, a comprehensive awareness campaign for the target group involving dental and medical healthcare providers was carried out as a model project [17]. As in the USA and England, the results showed that a structured awareness campaign improved the perception of tumours in the oral cavity [17–20].

However, as complex and multi-level prevention strategies for awareness campaigns must be planned and implemented in a context-sensitive manner [21–23], regional results only partially allow for national transfer. Therefore, possible professional groups involved in the early detection of oral cancer should be included from the beginning of concept development, and should also be considered important multipliers [24].

For the first time at a national level in Germany, this study aimed to assess the level of knowledge of diagnostic procedures and risk factors for oral cancer in order to develop a structured training programme.

## Methods

The study was conducted as an observational cross-sectional study, via an online survey, designed to assess at a single point in time the level of knowledge of the signs, symptoms, risk factors and early detection investigations for oral cancer. Furthermore, participants judged their overall knowledge level and capability to screen patients for oral cancer.

### Study population

The study population invited to the online survey comprised all dentists and maxillofacial surgeons in Germany (n = 72,468) who are obligatory members of one of the 17 German regional state Dental Associations (Supplement file 1). The three largest state Dental Associations are subdivided into numbers of district associations. The respective regional Dental Associations provided the number of members shown in Table 1.

**Table 1:** Distribution of members among the individual German regional state Dental Associations^a^

State Dental Association	Number of members
Baden-Wuerttemberg	9,250
Bavaria	12,033
Berlin	4,261
Brandenburg	1,946
Bremen	542
Hamburg	2,118
Hesse	5,765
Mecklenburg Western Pomerania	1,345
Lower Saxony	6,475
North Rhine	8,431
North Rhine-Westphalia	6.559
Rhineland-Palatinate	3,139
Saarland	701
Saxony	3,793
Saxony-Anhalt	1,816
Schleswig–Holstein	2411
Thuringia	1,883
**Total**	**72,468**

^a^Data from the German state Dental Association

The study included professionals who currently work in dentistry or as maxillofacial surgeons, regardless of their professional environment. Exclusion criteria were current non-clinical work, reported long-term illness, or parental leave.

### Survey

The questionnaire was developed and validated by the Yellowitz et al. working group [25, 26]. Members of the project group translated and validated the questionnaire into German according to international standards, and successfully used it in paper form for a regional project to improve the early detection of oral cancer [27].

For the present national study, the paper format had to be adapted to an online version without changing the content. Based on the German regional project results, questions on the number of patients per week and patients’ insurance status were removed.

Questions on the preference for the type of further continuing education were adapted to currently available formats, such as digital concepts.

After approval and finalisation within the working group, the online questionnaire comprised 38 items (Supplement file 2). This publication focused on the following sections:

Knowledge questions on signs, symptoms, and risk factors: This section consisted of several single- and one multiple-choice questions.

Opinions and continuing education: Questions about the participants’ knowledge and skills were answered on a four-point Likert scale ("strongly disagree"to"strongly agree"to"don't know").

Questions about continuing education were single-choice options: Preferences were rated on a four-point Likert scale ("very low"to"very high").

The section on socio-demographic variables (age, sex, professional qualification, professional environment, years in the profession) consisted of questions with suitable selections.

After finalising the questionnaire, the project group members conducted three test runs. These involved the technical implementation of the online version and correcting spelling and grammatical errors. The content of the items remained unchanged.

### Recruitment and study procedure

In order to conduct the online survey from October to December 2023, it was necessary to set up the national infrastructure together with the state Dental Associations. At the end of February 2023, at the invitation of the German Dental Association, the project leadership presented the project at a meeting of all state Dental Association presidents and requested participation. By the end of May 2023, all associations had agreed to participate and had sent the contact details of the administration, IT, and public relations departments. Subsequently, 18 national video conferences were held with these associations'contact persons to present and agree on the concept before the online survey's launch. The state Dental Associations had different membership numbers (Table 1) and thus had varying degrees of administrative staff and developed IT infrastructure to implement the recruitment process. In addition, not all associations had adequate email distribution lists of their members for the project. Thus, different approaches in the recruitment process were needed.

Based on the information collected, two dispatch variations were designed, one of which was divided into two sub-variations. Each association was then assigned to a respective variation, and the respective schedule was agreed upon with them.

The first variation included an article in the regional dental journal, the press organ for all regional dental associations in print format. These journals were posted once a month to once a quarter. Due to dental associations’ compulsory membership, every registered professional automatically receives this journal. The article provided information on the project and announced the online survey. Subsequently, all potential participants received an email or a personalised link via an online newsletter. After two and four weeks, a reminder was sent by email or online newsletter.

*For this personalised mailing*, each relevant association's administration received a list of personalised questionnaire links incorporating a unique pseudonym. The list of personalised links was sent to the association's member administrations to match it with the email addresses of potential participants. Survey answers were only associated with these pseudonyms and only available to the project group, not the dental associations. This approach ensured that neither the project group nor the association could track which members had participated in the online survey.

*In the second variation*, an open public link and a QR code were integrated into the article in the regional dental journal of the participating state Dental Association due to the lack of an online distribution list. Two weeks later, the reminder in the journal included a short article on the topic and another call for participation with the same link and QR code. Due to its size, one participating state Dental Association was further subdivided into district dental associations. In this case, the district Dental Associations issued two reminders after two and four weeks, and sent the articles with the public link and QR code via their online distribution.

### Statistical analysis

The data was examined for reliability, correctness and completeness before formal statistical analysis. In order to improve the reliability of the answers, we only included data from participants who spent at least 5 min and 50 s completing the survey (median time to complete the survey was 16 min and 40 s (Q1: 12 min and 26 s, Q3: 24 min and 19 s)). Furthermore, participants who responded in obvious patterns, which are considered signs of careless or otherwise problematic answers (e.g. always selecting the first option for a whole set of questions), were also excluded. The R package responsePatterns identified these patterns and flagged participants with autocorrelation > 0.95 within each section [28]. Finally, for each section of questions, only participants who answered at least two-thirds of the questions in the respective section were included in the statistical analysis.

Participants’ socio-demographic data and responses in the “opinion and continuing education” section were analysed descriptively, using numbers and percentages for categorical variables and mean, standard deviation, and range for continuous variables. For the"knowledge"section, across participants, the percentage of correct answers for each question was presented with 95% Clopper-Pearson confidence intervals. For each participant, an overall knowledge score as the percentage of correctly answered questions was calculated.

Linear regression analyses assessed the association between different characteristics or opinions of participants and their knowledge of oral cancer (knowledge score). Regressions with ordinal independent variables were conducted using equidistant quantitative variables, with higher values indicating high agreement. In scatterplots of these associations, dot sizes corresponded to observation numbers. In addition, statistical tests were conducted for the variables sex, years in profession (more relevant than age, but strongly correlated with it), time since last continuing education course on oral cancer, and the approbation of oral and maxillofacial surgeon. As sensitivity analyses, we also performed multiple linear regression analysis to obtain estimates for the effects of variables of interest while adjusting for the covariates sex, years in profession and time since last continuing education course on oral cancer. For each variable of interest, one such adjusted (multiple) model was calculated. Also, a model including all independent variables of interest was calculated. All performed statistical tests were two-sided at a significance level of 0.05. All statistical analyses were conducted using R version 4.3.3 [29].

## Results

### Participants

**Fig. 1:**
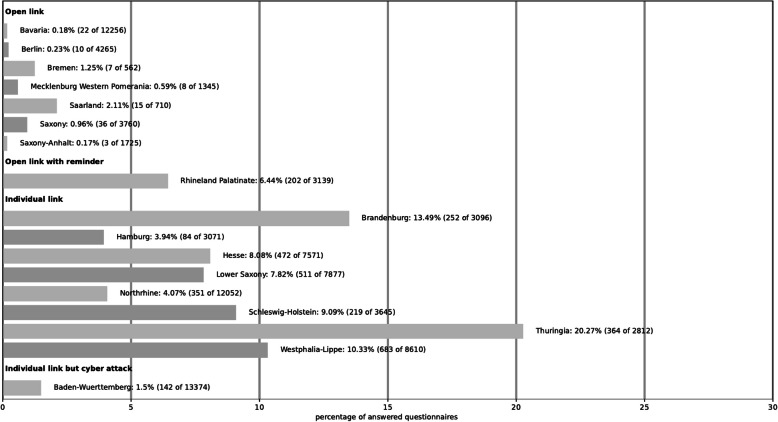
Participating members of the various state dental associations

Overall, 8,132 participants began the online survey. The participating members of the various state Dental Associations varied greatly due to the different dispatch variations, as described in Fig. 1. In addition, the Baden-Wuerttemberg Dental Association suffered a serious cyber-attack during the online survey.

After filtering for reliability and completeness, data from 3,458, 2,933 and 3,472 participants were available for the sections"socio-demographics","knowledge”, and “opinions and continuing education”, respectively.

Regarding the socio-demographic variables, most participants were women (almost 60%) (*n* = 2,060). Participants were between 20 and 69 years old, with the largest proportion aged 30 to 49. When asked about their professional environment, over 90% of the participants worked in private practice. The average number of years in the profession was around 21 years (± 11.9), ranging from 0 to 50 years. Table 2 shows the distribution of the study population.

**Table 2:** Demographic distribution of dentists and maxillofacial surgeons (*N* = 3,458)

Demographic	Number of cases (in %)
**Sex**	
Male	1,361 (39.4%)
Female	2,060 (59.6%)
Diverse	3 (0.1%)
Not known	34 (1.0%)
**Age**	
20–29	194 (5.6%)
30–39	853 (24.7%)
40–49	820 (23.7%)
50–59	912 (26.4%)
60–69	607 (17.6%)
Not known	72 (2.1%)
**Professional environment**	
Private practice	3,215 (93.0%)
Hospital	52 (1.5%)
University	69 (2.0%)
All other	122 (3.5%)
**Years of professional experience**	
Range	[0.0, 50.0]
Mean	20.9
Standard deviation	11.9
Not known	10 (0.3%)
**Approbation**	
Dentist	3,312 (95.39%)
Physician	1 (0.03%)
Dentist and physician (no oral and maxillofacial surgeon)	74 (2.13%)
Oral and maxillofacial surgeon	26 (0.75%)
Not known	45 (1.70%)

### Knowledge of diagnostic items

Overall, knowledge of various diagnostic aspects was good. Except for a few diagnostic items, the participants'knowledge was good to very good. About 95% of all participants were aware of the most important oral precursor lesion and knew the form of the tumour and while almost 90% knew the importance of early detection concerning the prognosis. However, only about three quarters knew that the main localisation was the tongue, and about 60% knew that those affected are asymptomatic in the early stages. Less than 60% knew the diagnostic signs of a lesion and an affected lymph node. The detailed presentation of all diagnostic items is shown in Table 3.

**Table 3:**
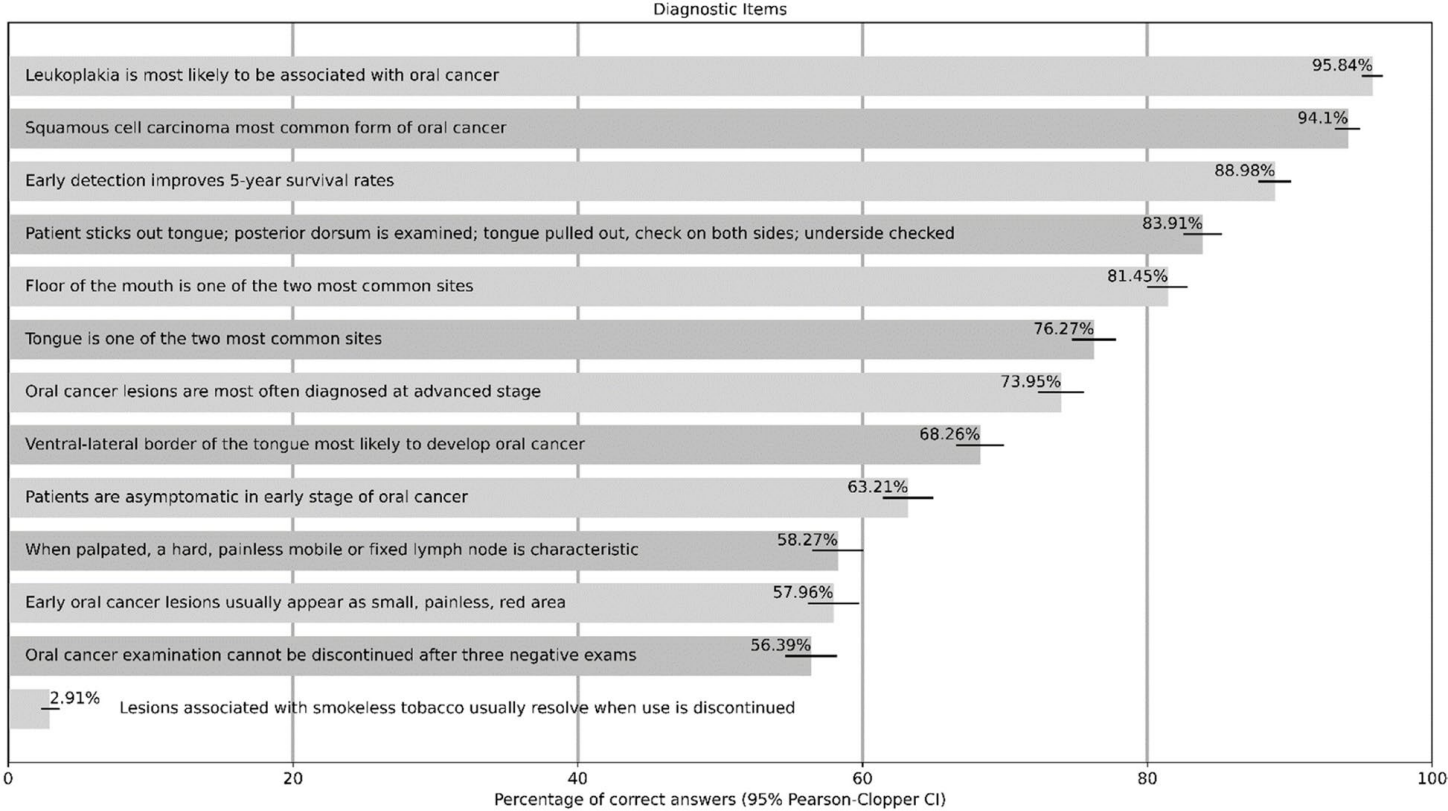
Participants’ knowledge of diagnostic procedures, signs and symptoms (*n* = 3,472)

### Knowledge of risk factors

Knowledge of the various possible risk factors was predominantly good. There was very good knowledge of the two main risk factors – tobacco and alcohol consumption (over 97%) – and also of prior oral cancer (96%). Over 80% of the participants knew that oral cancer is a tumour affecting older people, but only about 60% correctly chose the specific age range, i.e. 60 years and older. The detailed results of all the questions on risk factors are shown in Table 4.

**Table 4:**
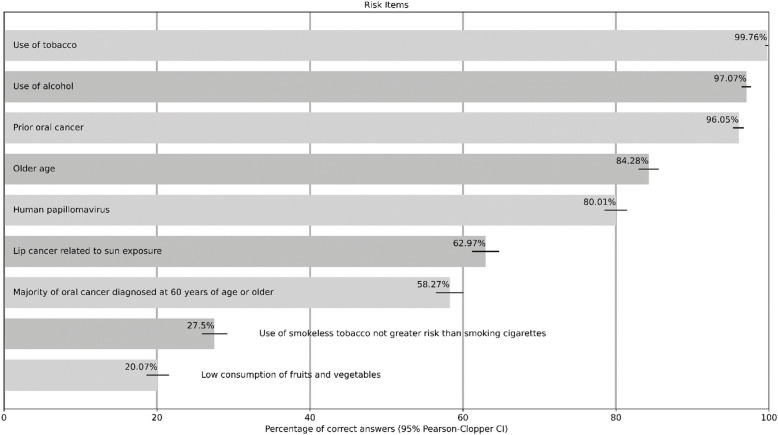
Participants’ knowledge of risk factors (*n* = 3,472)

Less professional experience or a shorter time since graduation was associated with significantly higher knowledge of diagnostic items and risk factors for oral cancer (*p* < 0.001). On average, participants with 10 years or less in the profession answered 1.42 percentage points more questions correctly. Women had slightly higher knowledge (*p* < 0.001) than men. Dentists and oral and maxillofacial surgeons did not have a significantly different level of knowledge (*p* = 0.8); on average dentists answered 63.8% of the questions correctly while oral and maxillofacial surgeons answered 62.2% correctly. However, due to the low number of oral and maxillofacial surgeons (*n* = 26), no definite conclusion can be drawn.

### Continuing education

An important aspect linked to the level of knowledge is participation in continuing education or interest in continuing education courses on this topic. There was a monotonic relationship between the time since the last continuing education course on oral cancer and participants'knowledge (*p* < 0.001). Those who attended a continuing education course on oral cancer within the last year had more accurate knowledge in this field and answered, on average, 2.12 percentage points more questions correctly than participants who had attended such a course more than five years ago.

More than half of the participants stated that they had attended training on this topic in the last five years (for a detailed distribution of time since the last continuing education course on oral cancer, see Supplement file 3), and more than 80% showed interest in continuing education courses. Regardless of who organised the training, the majority of participants preferred digital formats. Interestingly, however, the final question on the level of interest in various offers and sources of information on oral cancer showed that the highest level of interest, at around 80%, was in articles in specialist journals, followed by printed information materials at just over 70%. The detailed presentation is described in Table 5.

**Table 5:** Participants'interest in various offers and sources of information on oral cancer

Interest	Journal articles	Printed information sheets	E-learning on demand	Online information material	Personal exchange with colleagues
High	850 (24.5%)	699 (20.1%)	610 (17.6%)	739 (21.3%)	773 (22.3%)
Rather high	1,942 (55.9%)	1,771 (51%)	1,292 (37.2%)	1,603 (46.2%)	1,344 (38.7%)
Rather low	569 (16.4%)	831 (23.9%)	1,116 (32.1%)	861 (24.8%)	1,011 (29.1%)
Low	76 (2.2%)	136 (3.9%)	417 (12%)	234 (6.7%)	309 (8.9%)
Missing	35 (1%)	35 (1%)	37 (1.1%)	35 (1%)	35 (1%)

### Opinions

Participants also rated their overall level of knowledge of oral cancer. Only around 59% considered their level of knowledge to be current, but over 75% considered themselves adequately trained to perform the diagnostic examination, and only around 47% of participants recognised this competence in dentists in general. However, the majority (83%) of the professional group considered dentists, in general, to be qualified to carry out such an examination (Table 6).

**Table 6:** Participants’ opinion of competences (*N* = 3,472)

**My knowledge of oral cancer is current**	**Number of cases (in %)**
High	163 (4.7%)
Rather high	1,900 (54.7%)
Rather low	998 (28.7%)
Low	113 (3.3%)
Don't know	298 (8.6%)
**I am adequately trained to examine patients for oral cancer**	**Number of cases (in %)**
High	722 (20.8%)
Rather high	1,905 (54.9%)
Rather low	668 (19.2%)
Low	83 (2.4%)
Don't know	94 (2.7%)
**Most dentists are adequately trained to perform oral cancer exams**	**Number of cases (in %)**
High	254 (7.3%)
Rather high	1,411 (40.6%)
Rather low	1,200 (34.6%)
Low	257 (7.4%)
Don't know	350 (10.1%)
**Dentists are qualified to perform oral cancer examination**	**Number of cases (in %)**
High	1,201 (34.6%)
Rather high	1,691 (48.7%)
Rather low	444 (12.8%)
Low	75 (2.2%)
Don't know	61 (1.8%)

These stated opinions and judgements of qualification were associated with actual knowledge.

**Fig. 2:**
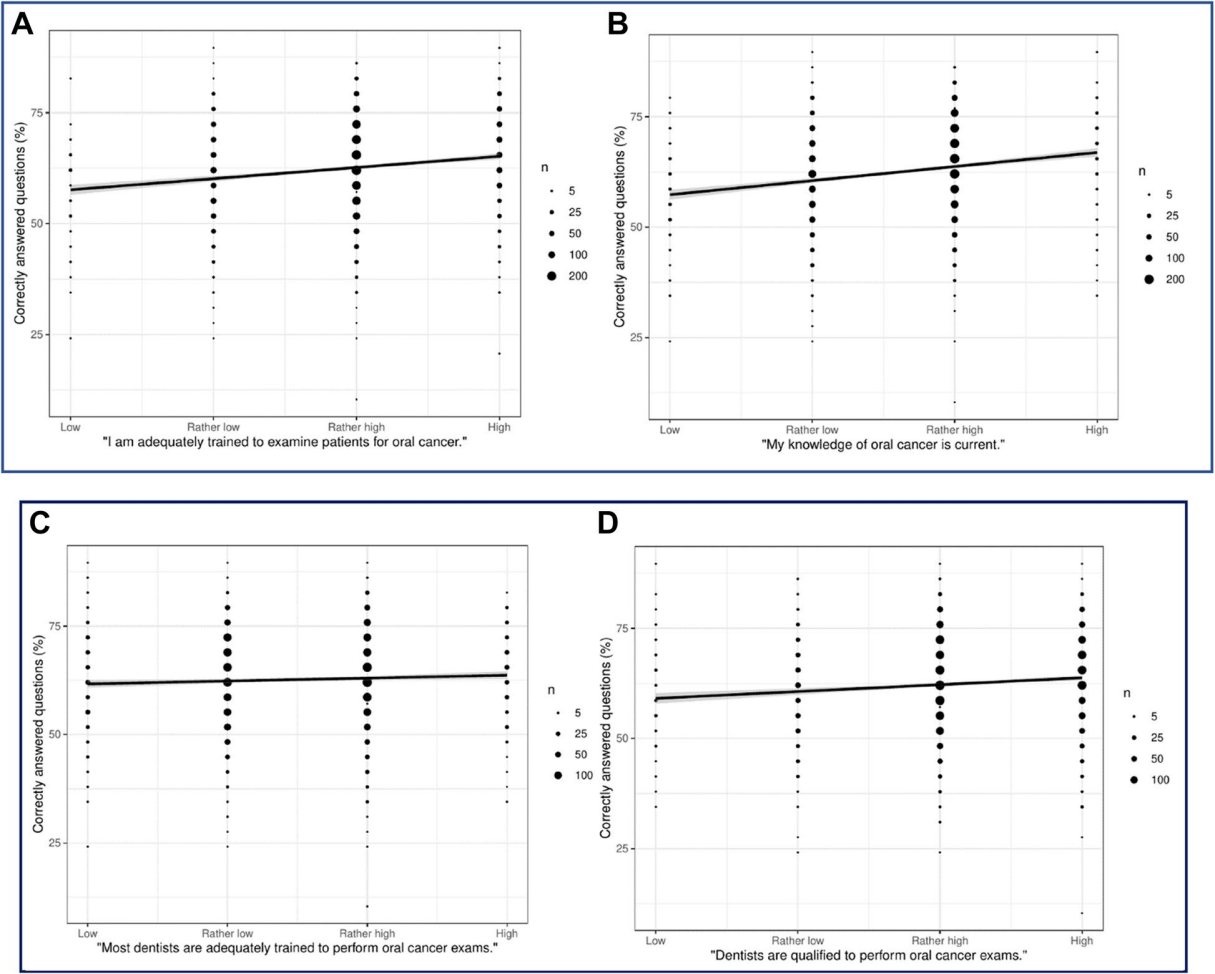
**A** and** B** Participants’ opinion of their competence regarding knowledge and training status. **C **and** D** Participants’ opinion of the competence of their professional group

The dentists’ self-assessment of knowledge and training correlated with their actual knowledge (*p* < 0.001 in both Figs. 2A and B). Those who considered their knowledge of oral cancer current (high or rather high agreement) scored on average 3.7 percentage points higher than those with low or rather low agreement.

The relationship between the participants’ knowledge and their perception of other dentists’ training or general qualifications for oral cancer examinations was also significant (*p* = 0.012 and *p* < 0.001, Figs. 2C and D). Here, the mean difference between (rather) high vs (rather) low agreement was 0.82 and 2.92 percentage points, respectively.

After adjusting for sex, years in the profession, and time since the last continuing education course on oral cancer, the relationships depicted in Figs. 2A to D remained nearly unchanged in terms of effect estimates and p-values. See Supplement file 4 for detailed information on multiple linear regression model coefficients and p-values. In line with the unadjusted analyses, also the adjusted analyses showed that the association of self-assessed knowledge with actual knowledge was strongest (R^2^ = 0.057, largest coefficient, *p* < 0.001), while the association of agreement with the statement “Most dentists are adequately trained to perform oral cancer exams” with actual knowledge was weakest but still statistically significant with our large sample size (R^2^ = 0.037, smallest coefficient, *p* = 0.046). In a multiple model with all variables of interest and the relevant covariates, all of these remained statistically significant.

## Discussion

Oral cancer continues to be a global health problem. An effective inspection of the oral cavity can and should be part of dental examinations. Dental healthcare providers thus play a very important role in the early detection of oral cancer. However, this requires knowledge of the symptoms, signs, risk factors, and preventive measures, i.e. the structured oral cavity examination [7]. In the following discussion, studies mainly published after 2010 using the same questionnaire were considered, ensuring both international comparability and the required quality criteria, especially the validity of the questionnaire. A literature review of other questionnaires did not clearly demonstrate these quality standards. Results of studies prior to 2010 were considered in a separate publication of the working group [30].

In our study, the participants'knowledge of the diagnostic items was heterogeneous, ranging from very good to a clear need for improvement. Very good knowledge (about 90%) was obtained for the most important oral precursor lesion, leukoplakia, and for the most common type of oral cancer, squamous cell carcinoma, compared to other studies, with > 70% from Eastern Europe [31] and < 30% in a study from the United Arab Emirates [32]. Furthermore, in our study and among participants in a study from Yemen, around 88% knew that early detection improves the five-year survival rate [33]. Studies from Jordan, West Bengal, Qatar and Indonesia also described a similar or slightly better level of knowledge, at 90% and more [9–11, 34, 35]. Regarding the two most common localisations – the tongue and floor of the mouth – there were interestingly very different levels of knowledge in our study and other studies. In our results, a slight difference in the level of knowledge in favour of the floor of the mouth was observed. These comparatively good values were also shown in the study from West Bengal [11]. In many cases, however, the knowledge level of this very important diagnostic aspect was significantly lower, even down to a third [12, 31, 34–36]. This heterogeneous picture of very good knowledge [9], down to around three-quarters in our study, and even to a level significantly below 50%, was also evident in the level of knowledge that those affected in the early stages have no symptoms [34, 35, 37]. This insufficient knowledge level was also observed in our study with regard to the question of how an early oral cancer lesion presents, compared to other studies with a third or less [10, 31, 34–37].

At well over 90%, our participants had a very good level of knowledge of the main risk factors – tobacco and alcohol consumption. Several other studies also found these very high values [9, 13, 33–35, 37, 39]. Interestingly, the level of knowledge of alcohol consumption as a risk factor was always slightly lower than tobacco consumption. A lower level of knowledge of both risk factors was observed in a study from Colombia [36], with around 78% for tobacco consumption and 57% for alcohol consumption, and a strikingly insufficient level of knowledge in a study from Yemen, with only 39% for both risk factors [12]. For risk factors prior to the development of oral cancer lesions, a high level of knowledge of over 90% was described in our study and others [9, 32, 34, 35, 38, 39], whereas some studies described these knowledge levels at around 75% to 90% [10, 13, 31, 33, 36].

As oral cancer is a tumour that affects older people, it is critical to have sufficient knowledge of the correct age group. Our participants showed a very good level of knowledge at over 80%. In several international studies, significantly lower values were observed, from around two-thirds [9, 13, 31, 32, 39], to around half [33, 35, 36, 38], to less than a third [10]. However, asking for the precise age group – 60 years and older – showed that the knowledge level dropped. Only around a third of our participants were able to answer the question correctly; in other studies, knowledge levels were below a third [12, 34, 35, 37].

Another underestimated risk factor was the relationship between lip cancer and sun exposure. In our study, only around two-thirds of the participants were aware of this, and studies from Yemen and Qatar also described this low level of knowledge [33, 34].

Although there was a sufficient to a very good level of knowledge for many questions in our study, only around half rated their knowledge level as current. This assessment was also reported in studies from Kuwait and West Bengal [9, 11]. In contrast, participants in studies from Yemen, Quartar and Palestine rated themselves higher [33, 34, 39]. However, significantly lower values were reported in studies from Sudan, Jordan and Congo [10, 12, 13].

The participants in our study and the studies from Kuwait and Yemen considered themselves adequately trained to examine their patients for oral cancer [9, 34], but their colleagues were not considered sufficiently trained. Here, the results in our study and those from Kuwait and Qatar were significantly lower [9, 34]. However, our study showed that this qualification was generally seen in this professional group.

An important question is whether there are factors that influence the level of knowledge. Our study showed a correlation with the number of years in the profession. The shorter the gap from the state exam, the higher the level of knowledge. This aspect is not surprising, and has also been observed in other studies: younger participants showed a higher level of knowledge, with the highest level present during the state exam [11, 12, 34, 35, 39]. Thus, the results showed a positive trend among participants in the survey, indicating that the relevant knowledge was imparted as part of the dental curriculum. A similar correlation was found between the time window of the last training or continuing education course and the level of knowledge, decreasing depending on how long ago the training took place. This correlation was also observed in the study by Wimardhani et al. (2021) and Shadid et al. (2023) [35, 39]. The Jboor et al. working group (2019) did not find this correlation in their participants [34].

The decline in knowledge over time emphasises the need for dental associations to continuously offer further training on this topic. Here, face-to-face events should be favoured, and online or hybrid formats should be used to increase participant interest. As the diagnosis of oral cancer is not regularly made in everyday practice and thus other dental topics may be of greater interest, it would be worth considering combining a discussion on oral cancer with other topics in one event. Furthermore, applications using artificial intelligence (AI) are increasingly receiving scientific attention in the field of early detection and could potentially support diagnostics for professionals. It remains to be seen where and in what form AI can be used, and also its limits [40, 41].

Overriding factors influencing knowledge levels must also be considered when comparing results internationally. For instance, the topic of oral cancer may be taught to varying degrees in the dental curriculum at universities. In addition, the working environment in the various healthcare systems and the associated opportunities for professional groups to perform regular preventative measures could also influence knowledge retention. These conditions could shape the extent to which these professional groups are offered further continuous training opportunities on this topic.

As in many other studies, this study has limitations, starting with the heterogeneous low response rate. The response rates for individual dental associations clearly showed that direct contact is much more effective. However, it was impossible to carry out this procedure at all dental associations due to IT restrictions, as participants may not be representative of the associations’ members of the various federal states. Furthermore, due to the flood of emails, individual selection must take place, and concerns about fake emails must also be considered. The length of the questionnaire might play a role, too.

Regarding the two professional oral healthcare groups, it would have been beneficial to analyse and present the survey data in a stratified manner. Unfortunately, this was impossible due to the low response rate of oral and maxillofacial surgeons.

Another aspect to be discussed is the questionnaire's focus on oral cancer. For future surveys, it would be preferable to integrate additional items that consider changes in age group and gender, and other elements regarding the diagnosis of oral precursor lesions.

A selection bias must be considered when interpreting the results, as the more committed, interested healthcare providers were potentially more likely to answer the questionnaire. Thus, the results cannot be regarded as representative of all healthcare providers in this sector.

One strength of the study is that the target group focused on practitioners in private practice, which was also reflected in the response rate. Even though the route we followed via the associations, combined with the online survey, was resource-consuming and had the disadvantages described above, it successfully reached the target group.

## Conclusions

This study shows that knowledge of localisation, signs, stage of diagnosis, and specific risk groups can be improved. These important aspects should be an integral part of the dental curriculum. The new dental licensing regulations in Germany, with a more explicit focus on prevention, offer the potential to better establish this in the curriculum. However, as the level of knowledge seems to decline over the years without continuous further training, it is recommended that institutions such as dental associations firmly establish the topic in their further training programme and offer it on an ongoing basis. Analogue and digital concepts should be used to increase the willingness of oral healthcare providers to undergo further training.

## Supplementary Information

Supplementary Material 1.

Supplementary Material 2.

Supplementary Material 3.

Supplementary Material 4.

## Data Availability

The datasets used and analysed in the current study are available from the corresponding author on reasonable request.
