# Supplementary material for: Knowledge level of diagnostic procedures and risk factors for oral cancer among oral healthcare providers in Germany
Source: BMC Oral Health. 2025 May 2;25:681. doi: 10.1186/s12903-025-06048-5 (PMC12048965; doi:10.1186/s12903-025-06048-5)
Supplement: Supplementary file 1 — Supplementary Material 1. [file 12903_2025_6048_MOESM1_ESM.pdf]

German regional state Dental Associations that collaborated in this study

(in alphabetical order)

The reginal state Dental Association of Baden-Wuerttemberg

The reginal state Dental Association of Bavaria

The reginal state Dental Association of Berlin

The reginal state Dental Association of Brandenburg

The reginal state Dental Association of Bremen

The reginal state Dental Association of Hamburg

The reginal state Dental Association of Hesse

The reginal state Dental Association of Mecklenburg-Western Pomerania

The reginal state Dental Association of Lower Saxony

The reginal state Dental Association of Northrhine

The reginal state Dental Association of North Rhine Westphalia

The reginal state Dental Association of Rhineland-Palatinate

The reginal state Dental Association of Saarland

The reginal state Dental Association of Saxony

The reginal state Dental Association of Saxony-Anhalt

The reginal state Dental Association of Schleswig-Holstein

The reginal state Dental Association of Thuringia
