# Supplementary material for: Knowledge level of diagnostic procedures and risk factors for oral cancer among oral healthcare providers in Germany
Source: BMC Oral Health. 2025 May 2;25:681. doi: 10.1186/s12903-025-06048-5 (PMC12048965; doi:10.1186/s12903-025-06048-5)
Supplement: Supplementary file 2 — Supplementary Material 2. [file 12903_2025_6048_MOESM2_ESM.pdf]

SURYEY OF DENTISTS PRACTICES AND OPIONNS ABOUT ORAL CANCER

Welcome to the online survey of dentists on the topic of oral cancer

as part of the project

"Improving the early detection of tumors of the oral cavity: Formative multi-level evaluation for the concept development of a national awareness campaign".

Your answers are important!

This survey contains 38 questions and takes about 15 minutes to complete.

Funded by

Realised by

Medical Faculty of the Christian-Albrechts-University Kiel (Project Leadership Prof. Dr. Katrin Hertrampf, Co-Leadership Prof. Dr. Astrid Dempfle)

Hanover Center for Health Communication, University of Music, Drama, and Media (Co-Leadership Prof. Dr. Eva Baumann)

Information on the project

As a member of a participating professional group, you are often the first to be confronted with tumors in the head and neck area in the course of your work. While the population is sensitized to changes in the external skin (e.g. due to melanomas), changes in the oral mucous often receive little attention. As a result, many of those affected unfortunately only consult a dentist or doctor for diagnosis when the disease is at an advanced stage. The start of treatment is often delayed, with the associated negative effects on the invasiveness of treatment, functional impairment and prognosis. In order to improve public awareness, personal responsibility and initiative with regard to this disease, we want to carry out a national prevention project. **It is important to us that you, as a participating professional group, are involved in this project from the outset.**

What is the current project about?

The project is designed on a voluntary basis. We would like to ask you to complete an online questionnaire on your level of knowledge of, for example, symptoms, signs and risk factors and your opinion and perspective on carrying out an oral mucosal examination.

Based on your information, we want to use the results for the development of a national training concept and make it available to you free of charge.

What happens to the information you provide in the online questionnaire?

The data from the online survey will be scientifically analyzed by the project group. Your participation will be pseudonymized by your dental association. The data from the survey will be collected by the project group under a study pseudonym and only published in the form of aggregated statistical analyses. Pseudonymization means that your personal data is encrypted. The Dental Association has no information as to whether you are taking part in the survey and will not receive the individual responses to the survey. The project group, in turn, has no information about your identity. You can withdraw from the study at any time without incurring any disadvantages. Even if you withdraw from the study, data can be used in a completely anonymized form. Persons who have access to the data are obliged to maintain data confidentiality.

For further information on the project management and the project, please click here (https://www.uksh.de/mkg- kiel/NaPrae_Mundkrebs).

Consent to participate in the project

I have been sufficiently informed about the aims and benefits of the study. I know that my participation in the study is voluntary and that I can withdraw my consent to participate at any time without giving reasons and without incurring any disadvantages.

By starting the online questionnaire, I agree that the data collected during the survey may be used pseudonymized for this study. I agree to the scientific analysis of the data and possible publication of the anonymous data.

I have the right to lodge a complaint with the Independent Centre for Data Protection Schleswig-Holstein in Kiel and the responsible state data protection officer (Ms Marit Hansen, tel. 0431-9881200).

I hereby give my voluntary consent to participate in this study.

☐ I agree and take part in this project.

(Button: Start of survey)

Dental practice*

In the following, we would like to ask you to answer questions about your patient structure and your assessment of the oral cancer examination. The term "oral cancer" used in the questionnaire stands for "cancers of the oral cavity and lip".

Please provide your best estimate of the percentage of your patients in the age groups for whom you provide an oral cancer examination at the INITIAL (emergency or scheduled) and RECALL appointments. If you do not provide oral cancer exams, please write "0".

Age	Initial Appt	Recall Appt
18 - 39 years	_________ %	_________%
40 and over	_________ %	_________ %

Please provide your best estimate of the percentage of your edentulous patients for whom you provide an oral examination. If you do not provide your edentulous patients with oral cancer examinations, please write 0.

____ % of edentulous patients

Please provide your best estimate of the percentage of your adult patients (18 years and older) for whom you routinely feel their necks to palpate their lymph nodes. If none please write 0.

_________ % of adult patients

In the past 12 months, in how many patients did you biopsy for suspicious oral lesions. If none please write “0“.

_________ of patients

*) The following parts of the questionnaire “Dental practice”, “Signs, Symptoms and Risk factors”, “Opinions” and “Health Histories” are originally developed by Yellowitz et al. (Yellowitz JA, Goodman HS: Assessing physicians’ and dentists’ oral cancer knowledge, opinions and practices. J Am Dent Assoc 1995; 126:53-60, Yellowitz JA, Horowith AM, Goodman HS, Canto MT, Farooq: Knowledge, opinions, and practices of general dentists regarding oral cancer: a pilot survey. J Am Dent Assoc 1998; 129:579-583).

In the past 12 months, how many patients did you refer for biopsy/diagnosis of a suspicious oral lesion:

If none please write “0“.

_________ of patients.

Signs, Symptoms and Risk Factors

Below we would like to ask you to answer questions about possible signs and symptoms of oral cancer and possible risk factors associated with oral cancer.

Excluding the lip, which of the following are the two most common sites of oral cancer:

(Check two)

1. ❑ Soft palate
2. ❑ Tongue
3. ❑ Gingiva
4. ❑ Buccal mucosa
5. ❑ Floor of mouth
6. ❑ Don’t know

The most common form of oral cancer is:

(Check only one)

1. ❑ Lymphoma
2. ❑ Squamous cell carcinoma
3. ❑ Basal cell carcinoma
4. ❑ Adenocarcinoma
5. ❑ Kaposi‘s sarcom
6. ❑ Don‘t know

Which one of the following factors is least likely to be associated with oral cancer:

(Check only one)

1. ❑ Increasing age
2. ❑ Familial clustering
3. ❑ Human papillomavirus (HPV)
4. ❑ Alcohol consumption
5. ❑ Tobacco use
6. ❑ Don't know

The symptom most commonly expressed by a patient with early oral cancer is:

(Check only one)

1. ❑ Pain
2. ❑ Ulceration
3. ❑ Swelling
4. ❑ None, patient is asymptomatic
5. ❑ Don't know

The majority of oral cancers are diagnosed in people who are:

(Check only one)

1. ❑ Less than 18 years of age
2. ❑ 18 - 39 years of age
3. ❑ 40 - 59 years of age
4. ❑ 60 years of age or alder
5. ❑ Don't know

A lymph node most characteristics of oral cancer metastasis, when palpated is:

(Check only one)

1. ❑ Hard, painful, mobile
2. ❑ Hard, painful, mobile or fixed
3. ❑ Soft, painful, mobile
4. ❑ Soft, painful, fixed of mobile
5. ❑ Don’t know

Which area of the tongue is most likely to develop oral cancer:

(Check only one)

1. ❑ All of the tongue
2. ❑ Dorsal surface
3. ❑ Ventral-lateral border
4. ❑ Anterior-lateral border
5. ❑ Base of tongue
6. ❑ None of the above
7. ❑ Don’t know

Oral cancer lesions are most often diagnosed in which stage:

(Check only one)

1. ❑ Premalignant
2. ❑ Early
3. ❑ Advanced
4. ❑ Don’t know

Lip cancers:

(Check only one)

1. ❑ Are related to sun exposure
2. ❑ Are increasing each year
3. ❑ Have a worse prognosis than most oral cancers
4. ❑ Affect the upper lip more frequently than the lower lip
5. ❑ Have not been related to any form of tobacco use
6. ❑ Don't know

Early oral cancer lesions usually appear as a:

(Check only one)

1. ❑ Small painless, red area
2. ❑ Small painful, read area
3. ❑ Small painful, white area
4. ❑ Small bleeding area
5. ❑ Don't know

When examining the tongue for oral cancer, the clinician should:

(Check only one)

1. ❑ Have patient stick out tongue as far as possible for inspection
2. ❑ Examine posterior dorsum of the tongue with a tongue blade or mirror
3. ❑ Pull the patient's tongue out and inspect both sides of it
4. ❑ Inspect the underside of the tongue by having the patient raise tongue
5. ❑ All of the above
6. ❑ Don't know

Of the following conditions, which one is most likely to be associated with oral cancer:

1. Leukoplakia
2. Pemphigus vulgaris
3. Migratory glossitis
4. Denture stomatitis
5. Don't know

Health Histories

When taking a health history, which of the following do you assess:

(Please select the appropriate answer for each item)

	(1) Yes	(2) No
1. Patient's past alcohol use………….	❑	❑
2. Patient's present alcohol use……	❑	❑
3. Type & amount of alcohol use………………	❑	❑
4. Patient's previous tobacco use………..	❑	❑
5. Patient's present tobacco use………….	❑	❑
6. Type & amount of tobacco…………..	❑	❑
7. Patient's history of cancer……..	❑	❑
8. Family history of caner………..	❑	❑

Which of the following factors places an individual at high risk for oral cancers:

(Please select the appropriate answer for each item)

	(1) Yes	(2) No	(3) Don't know
Older age…………………………….	❑	❑	❑
Use of alcohol………………………	❑	❑	❑
Use of tobacco products……..	❑	❑	❑
Family history of cancer………………….	❑	❑	❑
Low consumption of fruits and vegetables..	❑	❑	❑
Prior oral cancer lesion……….	❑	❑	❑
Poor fitting dentures……………	❑	❑	❑
Poor oral hygiene……………	❑	❑	❑
Use of spicy foods…………..	❑	❑	❑
Human papillomavirus……….	❑	❑	❑
Hot beverages & foods………	❑	❑	❑
Obesity………………………	❑	❑	❑

Opinions

In the following, we would like to ask you for your opinion on various aspects of oral cancer.

Please indicate the extent to which you agree or disagree with each of the following statements.

	Strongly Agree	Rather Agree	Rather Disagree	Strongly Disagree	Don‘t know
1. My knowledge of oral cancer is current..					
2. Oral cancer examinations for adults 40 years of age and older should be provided annually.					
3. Oral cancer examinations for adults 18-39 years af age should be provided annually					
4. I am comfortable referring patients with suspicious oral lesions to specialist...					
5. Oral cancer exams can be discontinued after 3 negative exams					
6. My patients are sufficiently knowledgeable about oral cancer risks factors					
7. My patients are sufficiently knowledgeable about oral cancer signs and symptoms					
8. Oral cancer examinations should be a separate reimbursable procedure					
9. I am comfortable palpating lymph nodes in neck of patients............					
10. The use of smokeless tobacco places a person at greater risk for oral cancer than those who smoke cigarettes................................					
11. Dentists are qualified to perform oral cancer exams..........................					
12. Physicians are qualified to perform oral cancer exams......................					
13. Early detection improves 5-year survival rates from oral cancers					
14. Lesions associated with smokeless tobacco generally resolve when use is discontinued					

Please indicate the extent to which you personally agree or disagree with each of the following statements

		Strongly Agree	Rather Agree	Rather Disagree	Strongly Disagree	Don't know
1.	I am adequately trained to provide tobacco cessation education					
2.	I am adequately trained to provide alcohol cessation education					
3.	Dentists should be trained to provide tobacco cessation education ...					
4.	Dentists should be trained to provide alcohol cessation education					
5.	I am adequately trained to examine patients for oral cancer					
6.	Most dentists are adequately trained to perform oral cancer exams					
7.	Most physicians are adequately trained to perform oral cancer exams					
8.	I am adequately trained to palpate lymph nodes in a patient's neck					

Continuing Dental Education

We would like to ask you to answer the following questions about your oral cancer continuing education.

When was the last time you attended a continuing education course on oral cancer:

1. ❑ Within the past year
2. ❑ During the past 2 – 5 years
3. ❑ More than 5 years ago
4. ❑ Never
5. ❑ Have yet to attend; graduated dental school within the last year
6. ❑ Don't know

Are you interested in attending continuing educational courses on oral cancer in the future?

1. ❑ Yes
2. ❑ Not sure/undecided
3. ❑ No

Which continuing educational course are you interested in? If you are interested, please indicate the preferred form of course.

Please select the appropriate answer for each item.

	Presence	Digital	Hybrid	Interest, but form doesn't matter	No Interest
Events organized by the associations	❑	❑	❑	❑	❑
Events organized by universities	❑	❑	❑	❑	❑
Events organized by professional associations	❑	❑	❑	❑	❑
Another event or form of realization, namely	______________				

How interested are you in the following offers and sources of information on the subject of oral cancer?

	very low	rather low	rather high	very high
Articles in specialized journals	❑	❑	❑	❑
Printed information materials	❑	❑	❑	❑
Online information materials	❑	❑	❑	❑
E-Learning on demand	❑	❑	❑	❑
Personal collegial exchange..	❑	❑	❑	❑

A different offer, namely ...... ______________

Personal Data

We would like to ask you to answer the following questions about demographics and your professional background.

Your age

Please enter your answer here:________

Your gender:

1. ❑ Male
2. ❑ Female
3. ❑ Diverse
4. ❑ Not specified

In which regional association do you work:

- ❑ Baden-Wuerttemberg
- ❑ Bavaria
- ❑ Berlin
- ❑ Brandenburg
- ❑ Bremen
- ❑ Hamburg
- ❑ Hesse
- ❑ Mecklenburg-Western Pomerania
- ❑ Lower Saxony
- ❑ North Rhine-Westphalia
- ❑ Rhineland-Palatinate
- ❑ Saarland
- ❑ Saxony
- ❑ Saxony-Anhalt
- ❑ Schleswig-Holstein
- ❑ Thuringia

Your current professional situation:

1. ❑ I work in private practice.
2. ❑ I work in a university dental clinic.
3. ❑ I work in a clinic
2. ❑ I do not work as a dentist.
3. ❑ Other:__________________

Number of years in the profession

Please enter your answer here:________

Your professional qualification:

1. ❑ Dental license
2. ❑ Medical license
3. ❑ Dental and medical license (maxillofacial surgeon)
4. ❑ Dental and medical license (no maxillofacial surgeon)

Is there anything else you would like to tell us about your experience with oral cancer or in relation to this survey?
If so, please write down your comments in the following
comment box below.

Thank you for completing this questionnaire.
We appreciate your co-operation and support in this project!

Submit your completed questionnaire:

Send
