# Supplementary material for: Knowledge level of diagnostic procedures and risk factors for oral cancer among oral healthcare providers in Germany
Source: BMC Oral Health. 2025 May 2;25:681. doi: 10.1186/s12903-025-06048-5 (PMC12048965; doi:10.1186/s12903-025-06048-5)
Supplement: Supplementary file 3 — Supplementary Material 3. [file 12903_2025_6048_MOESM3_ESM.pdf]

Time since last continuing education course on oral cancer of dentists and OMS* (N=3472)

Time	N (%)
Have yet to attend; graduated within the last year	85 (2.4%)
Within the past year	581 (16.7%)
During the past 2-5 years	1305 (37.6%)
More than 5 years ago	829 (23.9%)
Never	496 (14.3%)
Don’t know	142 (4.0%)
Not known/did not answer	34 (1.0%)

*) Oral and maxillofacial surgeons
