# Supplementary material for: Knowledge level of diagnostic procedures and risk factors for oral cancer among oral healthcare providers in Germany
Source: BMC Oral Health. 2025 May 2;25:681. doi: 10.1186/s12903-025-06048-5 (PMC12048965; doi:10.1186/s12903-025-06048-5)
Supplement: Supplementary file 4 — Supplementary Material 4. [file 12903_2025_6048_MOESM4_ESM.pdf]

Multiple linear regression models for correctly answered questions (%)

	Model 1			Model 2			Model 3			Model 4			Model 5			Model 6		
Predictors	*Estimates*	*CI*	*p*	*Estimates*	*CI*	*p*	*Estimates*	*CI*	*p*	*Estimates*	*CI*	*p*	*Estimates*	*CI*	*p*	*Estimates*	*CI*	*p*
(Intercept)	0.5976	0.5701 – 0.6251	**<0.001**	0.6090	0.5831 – 0.6349	**<0.001**	0.6686	0.6471 – 0.6901	**<0.001**	0.6344	0.6104 – 0.6584	**<0.001**	0.5670	0.5343 – 0.5996	**<0.001**	0.6826	0.6669 – 0.6982	**<0.001**
Number of years in profession	-0.0010	-0.0014 – -0.0007	**<0.001**	-0.0010	-0.0014 – -0.0007	**<0.001**	-0.0011	-0.0014 – -0.0008	**<0.001**	-0.0011	-0.0014 – -0.0008	**<0.001**	-0.0010	-0.0013 – -0.0006	**<0.001**	-0.0011	-0.0014 – -0.0008	**<0.001**
Sex: female	0.0102	0.0021 – 0.0184	**0.014**	0.0106	0.0026 – 0.0186	**0.010**	0.0085	0.0001 – 0.0168	**0.047**	0.0091	0.0011 – 0.0171	**0.025**	0.0122	0.0037 – 0.0206	**0.005**	0.0087	0.0007 – 0.0167	**0.034**
Time since last continuing education course on oral cancer	-0.0074	-0.0115 – -0.0034	**<0.001**	-0.0081	-0.0119 – -0.0042	**<0.001**	-0.0113	-0.0152 – -0.0074	**<0.001**	-0.0111	-0.0149 – -0.0074	**<0.001**	-0.0054	-0.0096 – -0.0012	**0.012**	-0.0122	-0.0160 – -0.0085	**<0.001**
Approbation: oral and maxillofacial surgeon																-0.0026	-0.0564 – 0.0513	0.925
My knowledge of oral cancer is current	0.0257	0.0189 – 0.0324	**<0.001**										0.0144	0.0061 – 0.0226	**0.001**			
I am adequately trained to examine patients for oral cancer				0.0198	0.0142 – 0.0254	**<0.001**							0.0167	0.0087 – 0.0247	**<0.001**			
Most dentists are adequately trained to perform oral cancer exams							0.0054	0.0001 – 0.0107	**0.046**				-0.0086	-0.0151 – -0.0020	**0.010**			
Dentists are qualified to perform oral cancer exams										0.0141	0.0089 – 0.0192	**<0.001**	0.0081	0.0020 – 0.0142	**0.009**			
Observations	2453			2590			2390			2611			2226			2656		
R^2 / R^2 adjusted	0.057 / 0.056			0.052 / 0.051			0.037 / 0.035			0.048 / 0.046			0.068 / 0.065			0.036 / 0.035		

Supplement file 4: Multiple linear regression models for number of correctly answered questions (%) as dependent variable
All models include an intercept term and the covariates (Number of year in profession, Sex, Time since last continuing education course on oral cancer). Models 1 to 4 include one additional independent variable of interest each, Model 5 includes all 4 additional independent variables, Model 6 includes the three covariates and approbation (oral and maxillofacial surgeon vs dentist).
